# The weight lowering effect of sibutramine and its impact on serum lipids in cardiovascular high risk patients with and without type 2 diabetes mellitus - an analysis from the SCOUT lead-in period

**DOI:** 10.1186/1472-6823-10-3

**Published:** 2010-02-26

**Authors:** Peter Weeke, Charlotte Andersson, Emil L Fosbøl, Bente Brendorp, Lars Køber, Arya M Sharma, Nick Finer, Philip T James, Ian D Caterson, Richard A Rode, Christian Torp-Pedersen

**Affiliations:** 1 Department of Cardiology, Gentofte Hospital, University of Copenhagen, Denmark; 2 Department of Cardiology, Glostrup Hospital, University of Copenhagen, Denmark; 3 Heart Centre, Rigshospitalet, University of Copenhagen, Denmark; 4 University of Alberta, Royal Alexandra Hospital, Edmonton, Alberta, Canada; 5 Addenbrooke's Hospital, Institute for Metabolic Science, Cambridge, UK; 6 London School of Hygiene and Tropical Medicine, London, UK; 7 Institute of Obesity Nutrition & Exercise, University of Sydney, NSW, Australia; 8 Abbott Laboratories, Abbott Park, Illinois, USA

## Abstract

**Background:**

Obesity, type 2 diabetes mellitus (T2D) and unhealthy blood lipid profile are strongly associated with the risk of developing cardiovascular disease (CVD). We examined whether blood lipid changes with short term administration of the weight lowering drug, sibutramine and lifestyle modification in obese and overweight high-risk patients was associated with T2D status at screening.

**Methods:**

The Sibutramine Cardiovascular OUTcomes (SCOUT) trial included obese and overweight patients at increased risk of cardiovascular events. All patients received guidance on diet and exercise plus once-daily 10 mg sibutramine during the 6-week, single blind lead-in period. Multivariable regression models were used to investigate factors associated with changes in lipid levels during the first four weeks of treatment.

**Results:**

A total of 10 742 patients received at least one dose of sibutramine during the 6-week lead-in period of SCOUT. After four weeks, patients experienced mean reductions in low density lipoprotein (LDL-C) 0.19 mmol/L, high density lipoprotein (HDL-C) 0.019 mmol/L, very low density lipoprotein (VLDL-C) 0.08 mmol/L, total cholesterol (TC) 0.31 mmol/L and triglycerides 0.24 mmol/L (p < 0.0001 for each). Four week changes in LDL-C, HDL-C and total cholesterol for patients without vs. with T2D were: LDL-C:-0.25 mmol/L vs. -0.18 mmol/L, P = 0.0004; HDL-C: -0.03 mmol/L vs. -0.02 mmol/L, P = 0.0014; total cholesterol: -0.37 mmol/l vs. -0.29 mmol/l, P = 0.0009. Multivariable regression analysis showed that similar decreases in body mass index (BMI) affected lipid changes differently according to diabetes status. A 1 kg/m^2 ^decrease in BMI in patients with T2D was associated with -0.09 mmol/L in LDL-C (P < 0.0001) and -0.01 mmol/L in HDL-C (P = 0.0001) but larger changes of -0.16 mmol/L LDL-C and -0.03 mmol/L in HDL-C (P < 0.0001 for both) in patients without T2D.

**Conclusion:**

Short term weight management with sibutramine therapy in obese or overweight high-risk patients induced significant mean reductions for all lipids. Those without T2D benefited most. Patients with hyperlipidaemia and the less obese patients also had greater falls in LDL-C and TC during weight loss. The trial is registered at ClinicalTrial.gov number: NCT00234832.

## Background

Obesity, hyperlipidaemia and type-2 diabetes mellitus (T2D) are well known risk factors for developing cardiovascular disease (CVD)[1,2]. It has been shown that a reduction in low-density lipoprotein cholesterol (LDL-C) reduces cardiovascular risk[3,4]. Reducing body mass index (BMI) in obese patients also reduces several risk factors associated to the development of CVD[2]. However, obese patients seldom manage to achieve and maintain weight loss through dietary changes and exercise alone[5]. The effects of weight loss and glycaemic control of T2D in terms of incidence of macrovascular disease are less clear[6]. To date no study has assessed the benefits of long term weight loss in high-risk patients. The Sibutramine Cardiovascular OUTcomes (SCOUT) trial included 10 742 obese or overweight high-risk patients where all patients received 10 mg sibutramine hydrochloride monohydrate (sibutramine) once daily for six weeks during the single-blind, lead-in phase of the study. This lead-in phase provides an opportunity to assess lipid changes during short term treatment with sibutramine and compare the responses in patients with and without T2D. This comparison provides preliminary data and highlights some of the variables to be considered in the ongoing randomized phase of the SCOUT trial.

## Methods

SCOUT is an ongoing, randomized, double-blind, placebo controlled, multicenter clinical study to assess the efficacy of sibutramine in reducing cardiovascular outcome in obese or overweight high-risk patients. During the 6-week, single-blind, lead-in period, all patients were treated with sibutramine 10 mg daily together with advice on diet and exercise (a 600 kcal/day deficit diet plan and an exercise program comprising >150 min of moderate exercise per week). Data presented in this paper are from the first four weeks of the 6-week SCOUT lead-in period before patients were randomized into the double-blind phase of the study.

Individuals eligible for inclusion in the study were men and women aged 55 years or older, with a BMI ≥ 27 kg/m^2 ^and ≥ 45 kg/m^2 ^or a BMI ≥ 25 kg/m^2 ^and <27 kg/m^2 ^with a waist circumference ≥ 102 cm (men) or ≥ 88 cm (women). Patients enrolled under the initial inclusion criteria were required to have diagnosed T2D together with at least one defined risk factor (hypertension, dyslipidaemia, current smoker, or diabetic nephropathy), or a history of coronary vascular disease (CVD), defined as coronary artery disease, peripheral arterial occlusive disease or stroke. A full description of the inclusion and exclusion criteria has been published previously[7]. Due to a lower than expected overall primary outcome rate, enrollment criteria were adjusted to amplify the recruitment of the highest risk patients 15 months after the first patient was enrolled. In particular, patients enrolled under the later restricted enrollment criteria were required to have both a history of CVD and a history of T2D with at least one additional risk factor.

Patients had a physical examination performed at the initial screening visit where information on body weight, vital signs, blood chemistry and haematology were obtained. Patients had follow-up visits every two weeks after the initial screening visit until the baseline visit, which was the start of the double-blind randomization period of the study. Patients who required an increase in their anti-hypertensive medication during this period were not to be randomized into the trial. At every follow-up visit, information on body weight and vital signs were obtained. Data on blood chemistry (fasting) and haematology were obtained at the screening visit and after four weeks of treatment. Some of the patients had a second haematology or a blood chemistry sample taken in relation to the screening visit or the visit after four weeks of treatment. For the present analysis, we used only the second blood samples if no data on the first sample were available. High density lipoprotein (HDL-C), low density lipoprotein (LDL-C), very low density lipoprotein (VLDL-C), total cholesterol (TC) and triglycerides were measured in mmol/L. HDL-C, VLDL-C, TC and triglycerides were measured directly in the blood samples in a certified central laboratory. LDL-C was estimated by Friedwald's equation when triglyceride levels were <4.52 mmol/L (calculated LDL-C cholesterol = [TC] - [HDL-C-cholesterol] - [triglycerides/5]), but was measured directly if triglyceride levels exceeded >4.52 mmol/L.

The trial is registered at ClinicalTrial.gov number: NCT00234832.

### Ethics

All participating patients gave informed written consent prior to participation. All approvals were obtained from relevant ethical committees and the study was performed in compliance with the Declaration of Helsinki.

### Statistics

Continuous variables were evaluated using both parametric (paired and two-sample t-tests) and non-parametric (Wilcoxon signed-rank and Wilcoxon rank sum tests) methods, with the latter providing similar results unless otherwise specified. Multivariable regression analysis was performed in order to identify factors associated with changes in lipid levels. Demographic and patient characteristics collected at the screening visit and listed in Table 1 were included as covariates in our analyses. When a significant two-way interaction was observed patients were stratified accordingly. Variables included in our final model were those with known or potential effects on the lipids of interest.

**Table 1:** Descriptive characteristics of enrolled patients

	NON-T2D	T2D
N	1748 (16.3%)	8981 (83.7%)
Age (years)	63.8 [0.14]	63.8 [0.07]
Male gender (%)	1148 (65.7%)	5063 (56.4%)
**Screening data and medication:**		
Weight (kg)	95.0 [0.35]	96.3 [0.16]
Waist Circumference men (cm)	112.5 [0.30]	114.8 [0.15]
Waist Circumference women (cm)	104.3 [0.46]	110.0 [0.18]
Hip Circumference men (cm)	110.8 [0.28]	112.6 [0.14]
Hip Circumference women (cm)	116.7 [0.45]	119.1 [0.19]
Body Mass Index (kg/m^2^)	33.4 [0.10]	34.6 [0.05]
Systolic Blood Presurre (mmHg)	136.3 [0.31]	138.6 [0.13]
Diastolic Blood Pressure (mmHg)	78.4 [0.20]	77.6 [0.09]
Pulse rate (b.p.m.)	68.0 [0.23]	71.7 [0.11]
LDL-C (mmol/L)	3.00 [0.02]	2.81 [0.01]
HDL-C (mmol/L)	1.25 [0.007]	1.18 [0.003]
VLDL-C (mmol/L)	0.86 [0.008]	0.95 [0.004]
TC (mmol/L)	5.13 [0.03]	5.01 [0.01]
Triglycerides (mmol/L)	1.98 [0.03]	2.31 [0.02]
Statins	73.0%	64.2%
Fibrates	5.7%	10.3%
Betablockers	69.1%	58.5%
ACEi	63.8%	79.4%
Smoking at screening	10.3%	9.6%
Drinking at screening	63.4%	53.5%
**Patient history of:**		
Stroke	9.2%	8.3%
Peripheral artery disease (PAD)	8.2%	11.4%
Coronary artery disease (CAD)	91.5%	60.8%
Acute Myocardial Infarction (AMI)	58.8%	37.1%
Congestive heart failure (CHF)	9.2%	8.4%
Hyperlipidemia	76.2%	81.8%
Hypertension	73.2%	90.7%

Presented are means with standard error of the mean within [].

13 patients with unknown risk category were excluded.

N denotes number of patients.

Dichotomous values are presented as percentages.

ACEi denotes angiotensin converting enzyme inhibitor.

Smoking at screening is defined as current tobacco use.

Drinking at screening is defined as at least 3 drinks per year.

All calculations were made using SAS^®^, version 9.1 (SAS Institute, Cary, North Carolina) on preliminary data available by September 2006. P-values ≤ 0.05 were regarded as statistically significant.

## Results

Included in the lead-in period of the SCOUT trial were 10 742 patients who took at least one dose of sibutramine. Patients with an unknown risk category were excluded (n = 13) as done previously[7]. Mean age and BMI for the study population were 63.8 years (Standard error of the mean (SEM) = 0.06) and 34.4 kg/m^2 ^(SEM = 0.04) respectively. Overall, patients experienced mean reductions in low density lipoprotein (LDL-C) (0.19 mmol/L), high density lipoprotein (HDL-C) (0.019 mmol/L), very low density lipoprotein (VLDL-C) (0.08 mmol/L), total cholesterol (TC) (0.31 mmol/L) and triglycerides (0.24 mmol/L) (P < 0.0001). Screening characteristics stratified according to diabetes status are presented in Table 1. The T2D group (n = 8981) comprised 83.7% of the overall study population. Of these 30.4% (n = 2734) were on treatment with insulin.

### Screening values

Mean LDL-C, HDL-C and TC at screening were found to be significantly greater in patients without T2D when compared to patients with T2D (Table 1) (P value for difference < 0.0001). However, mean VLDL-C and triglycerides at screening were significantly greater in patients with T2D than in patients without T2D (Table 1) (P for difference < 0.0001). Patients with T2D had a higher BMI than patients without T2D at screening (34.6 kg/m^2 ^vs. 33.4 kg/m^2^, P < 0.0001) (Table 1).

Overall 84.2% (n = 9033) of the patients had the metabolic syndrome according to the Adult Treatment Panel III defined criteria[8]. The majority (87.5%, n = 7901) of the patients with T2D met the criteria whereas patients without T2D usually did not (12.5%, n = 1132; p for difference < 0.0001).

### Changes in lipids and BMI

During the 4-week investigational period, all lipids were significantly decreased from screening values both in patients with and without T2D (P < 0.0001) (Table 2). Patients without T2D had greater decreases in LDL-C (P = 0.0004), HDL-C (P = 0.0014) and TC (P = 0.0009) levels than patients with T2D (Table 2).

**Table 2:** Changes in lipid variables over four weeks in patients with and without T2D

	non-T2D Mean changes	T2D Mean changes	p*
LDL-C (mmol/L)	-0.25 [-0.29, -0.21]	-0.18 [-0.20, -0.17]	0.0004
HDL-C (mmol/L)	-0.03 [-0.04, -0.02]	-0.02 [-0.02, -0.01]	0.0014
VLDL-C (mmol/L)	-0.09 [-0.11, -0.08]	-0.08 [-0.09, -0.07]	0.1564
TC (mmol/L)	-0.37 [-0.41, -0.33]	-0.29 [-0.31, -0.28]	0.0009
Triglycerides (mmol/L)	-0.22 [-0.27, -0.18]	-0.25 [-0.27, -0.22]	0.3231
BMI (kg/m^2^)	-0.81 [-0.84, -0.78]	-0.73 [-0.75, -0.72]	0.3887

In all instances, within-group changes from baseline were statistically significant (P < 0.0001)

Data presented as means and 95% Confidence intervals.

*P-values for difference in mean changes.

13 patients with unknown risk category were excluded.

**Figure 1:**
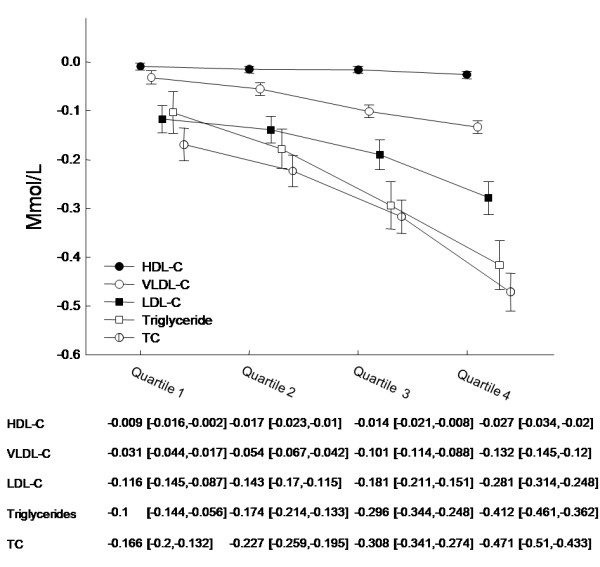
**Mean lipid changes in patients with T2D according to BMI reduction quartiles**. Lipid changes in patients with T2D stratified according to BMI reduction quartile (1-4). Quartile 1 represents the least reduction in BMI whereas quartile 4 represents the greatest. Mean lipid values are listed for each quartile with 95% confidence intervals. All values are measured in mmol/L. 13 patients with unknown risk category were excluded.

**Figure 2:**
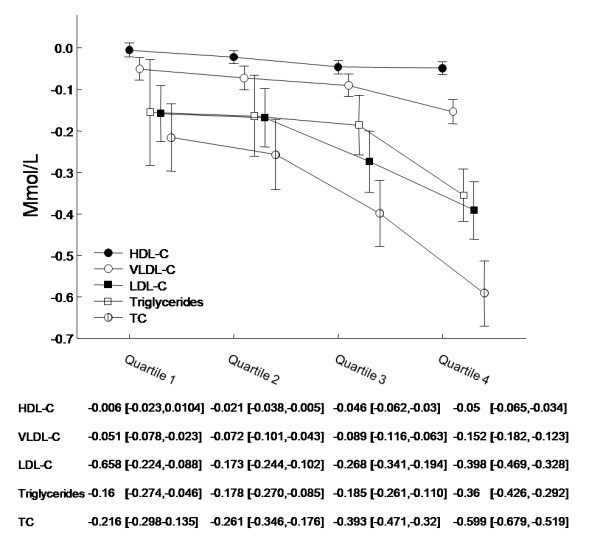
**Mean lipid changes in patients without T2D according to BMI reduction quartiles**. Lipid changes in patients without T2D stratified according to BMI reduction quartile (1-4). Quartile 1 represents the least reduction in BMI whereas quartile 4 represents the greatest. Mean lipid values are listed with 95% confidence intervals. All values are measured in mmol/L. 13 patients with unknown risk category were excluded.

Following four weeks of sibutramine treatment together with dietary advice and exercise, mean BMI was reduced by 0.75 kg/m^2 ^in patients who had measurements at both time points. Further, we found patients without T2D had greater mean decreases in BMI than patients with T2D (0.81 kg/m^2 ^vs. 0.74 kg/m^2^, P < 0.0001). Figures 1 and 2 depict the relationship between the magnitude of BMI reduction (quartiles) following four weeks of treatment and the corresponding reductions in lipids, with reductions in BMI producing greater lipid level reductions overall.

**Figure 3:**
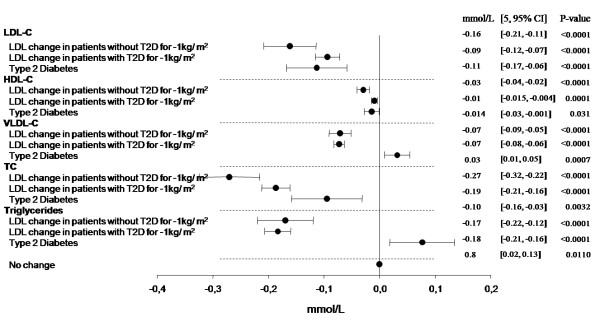
**The effects of T2D and 1 kg/m^2 ^BMI-decrement in patients with/without T2D on predicted lipid changes**. Multivariable regression analysis demonstrating the effects of a 1 kg/m^2 ^BMI decrement in patients with/without T2D and the effect of T2D on predicted lipid changes. The model is adjusted for all covariates in Table 1. 13 patients with unknown risk category were excluded.

Results from multivariable regression models including all covariates from Table 1 are listed in the Additional File 1. Notably, we found that a reduction in BMI of 1 kg/m^2 ^affected the change in LDL-C, HDL-C and TC differently depending on the patient's diabetes status (LDL-C: P for interaction 0.0086; HDL-C: P for interaction 0.0018; TC: P for interaction 0.0055). Therefore, we stratified the change in BMI according to patient diabetes status (Figures 1, 2 and 3). BMI reductions appeared to influence triglyceride and VLDL-C similarly in patients with and without T2D (Fig 3; triglycerides: P for interaction 0.6251; VLDL-C: P for interaction 0.8734). No interaction was found for T2D with gender or age (data not shown).

Presented in Figure 3 are the effects of a positive T2D status and the effects of a decrement in BMI (1 kg/m^2^) stratified according to T2D status on predicted changes in lipids from the multivariable regression models. The effect of a BMI decrement (1 kg/m^2^) in patients without T2D produced greater decreases in LDL-C, HDL-C and TC levels than a similar BMI decrement in patients with T2D (Figure 3). Also illustrated in Figure 3 are the effects of T2D status on lipid changes. A positive T2D status predicted greater mean reductions in LDL-C (0.11 mmol/l, P < 0.0001), HDL-C (0.014 mmol/l, P = 0.031), and TC (0.10 mmol/l, P = 0.0032), but lesser mean reductions in VLDL-C (0.03 mmol/l, P = 0.0007) and triglycerides (0.8 mmol/l, P = 0.0110), when all other factors are accounted for (Figure 3).

### Other results

In our multivariable regression analyses with information on screening levels for examined lipids, we found male gender to be significantly associated with reductions in all examined lipids (LDL-C 0.69 mmol/L; HDL-C 0.02 mmol/L; VLDL-C 0.05 mmol/L; TC 0.13 mmol/L and triglycerides 0.09 mmol/L) (Additional File 1). A history of smoking was associated with lesser mean reductions in VLDL-C, TC and triglycerides (Additional File 1). The use of statins was found to help induce significantly greater decreases in LDL-C (0.30 mmol/L) and TC (0.31 mmol/L), but was not significantly associated with selective effects on HDL-C and triglycerides. Furthermore, the use of fibrates showed significantly higher values for VLDL-C (0.02 mmol/L) and triglycerides (0.08 mmol/L) once the other covariates had been accounted for (Additional File 1). No interaction was found with respect to the change in VLDL-C, (P for interaction 0.9044) or triglycerides, (P for interaction 0.9095) when both the use of fibrates and the degree of weight loss was considered.

## Discussion

Obese and overweight high-risk patients with and without T2D experienced mean reductions in LDL-C, HDL-C, VLDL-C, TC and triglycerides following four weeks of weight loss therapy with sibutramine and lifestyle changes (Table 2). Patients without T2D showed the greatest mean reductions in LDL-C, HDL-C and TC (Table 2). We found that T2D status had a significant influence on all lipids following four weeks of sibutramine treatment during the 6-week lead-in period of the study (Figure 3). Furthermore, T2D status also induced different effects on LDL-C, HDL-C and TC levels for each 1 kg/m^2 ^decrement in BMI (Figure 3).

Overall, average LDL-C levels decreased by 7.3% in patients with and without T2D during the treatment period. There is a strong causal link between elevated levels of LDL-C and the development of atherosclerotic vascular disease [9-13]. Obese, overweight high-risk and abdominally obese patients are often susceptible to develop an adverse lipid profile also known as the 'lipid triad' (elevated LDL-C, low HDL-C, elevated triglycerides) [14] which is one of several contributing risk factors for developing CVD. Thus, it is evident that lowering LDL-C levels is an important step in combating their increased risk of CVD[15]. We saw a greater mean LDL-C reduction in patients without T2D than in patients with T2D. These findings are to some extent related to the higher LDL-C screening values observed in patients without T2D than in patients with T2D (3.0 mmol/L vs. 2.8 mmol/L respectively, P < 0.0001). Also, this can relate to the well known fact that individuals prone to develop cardiovascular disease have higher TC and LDL-C levels and, for a standard reduction in saturated fat intake, their responsiveness to a standardized dietary change is greater[16]. Further, patients without T2D experienced greater reductions in BMI than patients with T2D, thus amplifying the observed decreases in TC and LDL-C (Figure 1 and 2). This smaller weight loss in patients with T2D has been well documented but the reasons for the differences in response remain obscure[17]. The use of statins was associated with greater reductions in LDL-C in both patients with and without T2D (Additional File 1) which implies an interactive effect of stains with dietary changes and sibutramine treatment. However, the use of statins was significantly greater among patients without T2D than with T2D (73.0% vs. 64.2% respectively, P < 0.0001) which could contribute to the greater mean LDL-C reduction found in patients without T2D. These three features namely the greater fall in BMI, the use of statins and the greater hyperlipidaemia, therefore, seemed to have combined to induce the greater fall in TC and LDL-C in the patients without T2D.

HDL-C, often labeled as being a 'good cholesterol' because of its protective effects against the development of CHD [18,19], is known to decrease during an acute, non-steady state phase of weight loss, but then increases above baseline levels following long term weight loss maintenance[20]. In this SCOUT trial overall HDL-C levels fell by 1.4% on average as a result of four weeks of weight management with sibutramine, dietary advice and exercise. No increase in HDL-C levels following four weeks of sibutramine therapy has been reported previously[21]. The Sibutramine Trial of Obesity Reduction and Maintenance (STORM) was, however, a longer-term two year study which assessed the effect of sibutramine as a pharmacological adjunct to dietary advice and exercise. Sibutramine was found to induce significant increases in HDL-C levels following six months of therapy[22,23] but no acute changes during the initial period of weight loss. The study also suggested that the HDL-C increases were greater than expected for weight loss alone, and identified sibutramine as an inducer of HDL-C independent of the effects of weight changes[23]. Thus, our results on HDL-C are concordant with previously described short term responses where an initial decrease in HDL-C is expected. The long term results will be available after SCOUT ends in 2009.

According to our multivariable regression models, the effects of a BMI decrement (1 kg/m^2^) in patients with T2D predicts a 0.01 mmol/l greater reduction in triglyceride levels than equivalent BMI reductions in patients without T2D (Figure 3) whereas the VLDL-C changes were similar (Figure 3). These changes may reflect the impact of changes in dietary composition and/or nutrient intake as well as an effect of sibutramine-amplified weight loss in improving insulin sensitivity in the insulin-resistant T2D patients. However, the changes are dominated by factors other than the presence of T2D because the actual 4-week mean changes show no significant difference in decreases for triglyceride and VLDL-C in patients with and without T2D (Table 2).

According to our multivariable regression analyses, the use of fibrates was associated with higher levels of triglyceride and VLDL-C levels (Additional File 1). However, the clinical decision to use fibrates may reflect a greater CVD problem in these patients and therefore confounds the interpretation of the changes observed. Our findings on triglyceride reductions are similar to the previously reported findings with sibutramine in the STORM study[23], but the reduced relative and absolute risk for CVD following reductions in triglyceride levels is not as great as the benefit of lower LDL-C levels[24].

A high HDL-C/LDL-C ratio is predictive of a reduced incidence of CVD. The mean HDL-C/LDL-C ratio at screening was 0.46 for patients without T2D and 0.48 for patients with T2D. HDL-C/LDL-C ratios were seen in both patients with (0.51) and without T2D (0.49) following four weeks of treatment with sibutramine and lifestyle changes. Thus, despite the expected initial reductions in HDL-C, the overall increases in the HDL-C/LDL-C ratios were favorable and reflect the greater decreases in LDL-C levels.

The multivariable regression analyses showed that patients with T2D did reduce their LDL-C and HDL-C levels with weight loss. Nevertheless these patients with T2D had marked dyslipidaemia which was one of the risk requirements in the recruitment of the T2D patients for this trial. The relatively early adjustment of patient selection also amplified the proportion of T2D patients who already had cardiovascular events. Thus it is not surprising that dyslipidaemia rates were high and that there was a fall in LDL-C levels with weight loss following dietary adjustment and exercise with sibutramine as a pharmacological adjunct. The dietary advice with diet sheets specified a marked reduction in saturated fatty acid intake and an increase in dietary n-3 fatty acids. The data do emphasize the value of taking a proactive approach to weight reduction in patients with T2D despite their resistance to weight loss and the commonly held approach that one should concentrate on the co-morbidities of obesity in T2D management rather than tackling the weight loss per se.

### Limitations to this study

The data presented in this paper are from the lead-in period of the SCOUT trial covering four weeks of sibutramine treatment with all patients receiving sibutramine 10 mg daily as a test to their possible sensitivity to the drug. Therefore we cannot make conclusions relating to any specific effects of sibutramine treatment and lipid changes. Further, there were no dose changes either to allow any dose response analyses. Thus, we are unable to distinguish the effect induced by sibutramine alone from the effects caused by dietary advice and exercise.

Patients were requested to fast for every visit that included blood samples and we have specific statements from the patients that they complied. However, patient visits were carried out at different times during the day; for example, if the first visit was in the morning and the second in the late afternoon, this could potentially influence measured lipid levels. Thus, the lipid profile may have been influenced by the varying times of venous sampling.

Regression towards the mean is a problem we have tried to overcome by including our initial lipid screening values as explanatory variables for all analyses. Further, potential two-way interactions (i.e., stratification-by-baseline factor) were evaluated in an attempt to evaluate changes in the dependent variable for potential regression towards the mean (data not shown).

Notably, the SCOUT trial was not specifically designed to compare lipid changes following four weeks of treatment with sibutramine as an adjunct to dietary advice and exercise which is a limitation.

LDL-C levels were either measured directly or indirectly (by calculation). If triglyceride levels exceeded >4.52 mmol/L then LDL-C levels were measured directly. The use, which is common practice, of an estimated LDL-C gives some uncertainty compared to direct measurement.

## Conclusions

Our study shows that short-term sibutramine therapy with dietary advice and exercise in obese or overweight high risk patients with and without T2D is associated with overall improvements in lipid profiles. LDL-C levels decreased significantly in patients with and without T2D. Patients without T2D experienced greater decreases in LDL-C and TC for each 1 kg/m^2^decrement in BMI than patients with T2D. HDL-C levels decreased, but these findings may be attributed to the acute effects of weight loss during the short duration of the study. Hyperlipidaemia at screening also predicted greater benefit from the weight loss measures but whether the changes in BMI and lipids translate into a clinical benefit will be further clarified when the SCOUT trial ends. Our preliminary results are consistent with the use of sibutramine treatment with dietary advice and exercise to improve the treatment of obesity and overweight patients regardless of diabetes status. Further studies are required to confirm these findings in this patient population.

## Competing interests

Peter Weeke, Charlotte Andersson, Emil Loldrup Fosbøl, Bente Brendorp and Lars Køber declare no conflict of interests.

Ian D. Caterson: Investigator for clinical trials on obesity for Servier Laboratories, 3 M Pharmaceuticals, GSK, Metabolic Pty Ltd, MSD, Pfizer, Roche Products and sanofi-aventis; consultant and speaker at meetings organized by medical societies globally paid for by Abbott and sanofi-aventis; consultant for Roche Products, Abbott and sanofi-aventis; member of SCOUT ESC receiving payment from Abbott (honoraria/travel expenses).

Nicholas Finer: Consultant for Novartis, Shionogi, Merck, Abbott, sanofi-aventis, Amylin Pharmaceuticals Ajinomoto and GSK; received lectureship fees from Abbott, sanofi-aventis, Roche and Novo-Nordisk; received grant support from Merck, Novartis, Roche, Alizyme, Pfizer, Johnson and Johnson, Abbott and sanofi-aventis; member of the SCOUT ESC receiving payment from Abbott (honoraria/travel expenses).

Philip T. James: Consultant and speaker at meetings organized by medical societies globally, paid for by Abbott and sanofi-aventis; chairman of the SCOUT ESC receiving payment from Abbott (honoraria/travel expenses); member of the sanofi-aventis international advisory group; trustee of the IASO charity, which receives grants from Abbott, GSK, Merck, Pfizer, Roche, and sanofi-aventis.

Arya M. Sharma: Consulting/speaker honoraria and research support from Abbott, sanofi-aventis, Boeringer-Ingelheim, Novartis, Johnson & Johnson; member of SCOUT ESC receiving payment from Abbott (honoraria/travel expenses).

Christian Torp-Pedersen: Member of the SCOUT ESC receiving payment from Abbott (honoraria/travel expenses); received honoraria (<$10,000 US total) for participation on Steering Committee and/or Advisory Boards for anti-arrhythmic drugs and/or cardiac diseases; Data Safety Monitoring Board member for growth hormone trial.

Richard A. Rode: Employee of, and stockholder in Abbott.

**Source of support**: Abbott Laboratories provided funding for all aspects of this clinical study.

## Authors' contributions

PW: Statistical analysis of the data and wrote the paper. CA, ELF, and BB: Critical revision of manuscript. LK, AMS, NF, PTJ, IDC and CTP: Conceived and designed the experiments. Critical revision of the manuscript. RAR: Statistical analysis of the data. All authors have read and approved the final version of the manuscript.

## Pre-publication history

The pre-publication history for this paper can be accessed here:

http://www.biomedcentral.com/1472-6823/10/3/prepub

## Supplementary Material

Additional file 1 **Estimated effect of variables from Table **1**on lipid changes according to multivariable regression analyses**. The additional file contains the results from the multivariable regression analysis examining the effects on lipid changes when controlling for all variables listed in Table 1 Click here for file

## References

[B1] Fox CS Pencina MJ Wilson PW Paynter NP Vasan RS D'Agostino RB Sr Lifetime risk of cardiovascular disease among individuals with and without diabetes stratified by obesity status in the Framingham heart study Diabetes Care 2008 31 1582 1584 10.2337/dc08-0025 18458146 PMC2494632

[B2] Poirier P Giles TD Bray GA Hong Y Stern JS Pi-Sunyer FX Eckel RH Obesity and cardiovascular disease: pathophysiology, evaluation, and effect of weight loss: an update of the 1997 American Heart Association Scientific Statement on Obesity and Heart Disease from the Obesity Committee of the Council on Nutrition, Physical Activity, and Metabolism Circulation 2006 113 898 918 10.1161/CIRCULATIONAHA.106.171016 16380542

[B3] Flegal KM Graubard BI Williamson DF Gail MH Cause-specific excess deaths associated with underweight, overweight, and obesity JAMA 2007 298 2028 2037 10.1001/jama.298.17.2028 17986696

[B4] Haslam DW James WP Obesity Lancet 2005 366 1197 1209 10.1016/S0140-6736(05)67483-1 16198769

[B5] Mauro M Taylor V Wharton S Sharma AM Barriers to obesity treatment Eur J Intern Med 2008 19 173 180 10.1016/j.ejim.2007.09.011 18395160

[B6] Holman RR Paul SK Bethel MA Matthews DR Neil HA 10-year follow-up of intensive glucose control in type 2 diabetes N Engl J Med 2008 359 1577 1589 10.1056/NEJMoa0806470 18784090

[B7] Torp-Pedersen C Caterson I Coutinho W Finer N Van Gaal L Maggioni A Sharma A Brisco W Deaton R Shepherd G James P Cardiovascular responses to weight management and sibutramine in high-risk subjects: an analysis from the SCOUT trial Eur Heart J 2007 28 2915 2923 10.1093/eurheartj/ehm217 17595194

[B8] Third Report of the National Cholesterol Education Program (NCEP) Expert Panel on Detection, Evaluation, and Treatment of High Blood Cholesterol in Adults (Adult Treatment Panel III) final report Circulation 2002 106 3143 3421 12485966

[B9] Graham I Atar D Borch-Johnsen K Boysen G Burell G Cifkova R Dallongeville J De Backer G Ebrahim S Gjelsvik B European guidelines on cardiovascular disease prevention in clinical practice: executive summary Eur Heart J 2007 28 2375 2414 10.1093/eurheartj/ehm316 17726041

[B10] Liu J Sempos CT Donahue RP Dorn J Trevisan M Grundy SM Non-high-density lipoprotein and very-low-density lipoprotein cholesterol and their risk predictive values in coronary heart disease Am J Cardiol 2006 98 1363 1368 10.1016/j.amjcard.2006.06.032 17134630

[B11] Wilson PW Bozeman SR Burton TM Hoaglin DC Ben-Joseph R Pashos CL Prediction of first events of coronary heart disease and stroke with consideration of adiposity Circulation 2008 118 124 130 10.1161/CIRCULATIONAHA.108.772962 18591432

[B12] Halle M Berg A Frey I Konig D Keul J Baumstark MW Relationship between obesity and concentration and composition of low-density lipoprotein subfractions in normoinsulinemic men Metabolism 1995 44 1384 1390 10.1016/0026-0495(95)90134-5 7476322

[B13] De Backer G Ambrosioni E Borch-Johnsen K Brotons C Cifkova R Dallongeville J Ebrahim S Faergeman O Graham I Mancia G European guidelines on cardiovascular disease prevention in clinical practice. Third Joint Task Force of European and Other Societies on Cardiovascular Disease Prevention in Clinical Practice Eur Heart J 2003 24 1601 1610 10.1016/S0195-668X(03)00347-6 12964575

[B14] Ballantyne CM Olsson AG Cook TJ Mercuri MF Pedersen TR Kjekshus J Influence of low high-density lipoprotein cholesterol and elevated triglyceride on coronary heart disease events and response to simvastatin therapy in 4S Circulation 2001 104 3046 3051 10.1161/hc5001.100624 11748098

[B15] Bogers RP Bemelmans WJ Hoogenveen RT Boshuizen HC Woodward M Knekt P van Dam RM Hu FB Visscher TL Menotti A Association of overweight with increased risk of coronary heart disease partly independent of blood pressure and cholesterol levels: a meta-analysis of 21 cohort studies including more than 300 000 persons Arch Intern Med 2007 167 1720 1728 10.1001/archinte.167.16.1720 17846390

[B16] Brunner EJ Thorogood M Rees K Hewitt G Dietary advice for reducing cardiovascular risk Cochrane Database Syst Rev 2005 CD002128 23543514 10.1002/14651858.CD002128.pub4

[B17] Finer N Ryan DH Renz CL Hewkin AC Prediction of response to sibutramine therapy in obese non-diabetic and diabetic patients Diabetes Obes Metab 2006 8 206 213 10.1111/j.1463-1326.2005.00481.x 16448525

[B18] DeFaria Yeh D Freeman MW Meigs JB Grant RW Risk factors for coronary artery disease in patients with elevated high-density lipoprotein cholesterol Am J Cardiol 2007 99 1 4 10.1016/j.amjcard.2006.07.053 17196452

[B19] Jeppesen J Hein HO Suadicani P Gyntelberg F Triglyceride concentration and ischemic heart disease: an eight-year follow-up in the Copenhagen Male Study Circulation 1998 97 1029 1036 9531248 10.1161/01.cir.97.11.1029

[B20] Schieffer B Moore D Funke E Hogan S Alphin F Hamilton M Heyden S Reduction of atherogenic risk factors by short-term weight reduction. Evidence of the efficacy of National Cholesterol Education Program guidelines for the obese Klin Wochenschr 1991 69 163 167 10.1007/BF01665860 2041377

[B21] Shechter M Beigel R Freimark D Matetzky S Feinberg MS Short-term sibutramine therapy is associated with weight loss and improved endothelial function in obese patients with coronary artery disease Am J Cardiol 2006 97 1650 1653 10.1016/j.amjcard.2005.12.059 16728231

[B22] Dujovne CA Zavoral JH Rowe E Mendel CM Effects of sibutramine on body weight and serum lipids: a double-blind, randomized, placebo-controlled study in 322 overweight and obese patients with dyslipidemia Am Heart J 2001 142 489 497 10.1067/mhj.2001.117510 11526363

[B23] James WP Astrup A Finer N Hilsted J Kopelman P Rossner S Saris WH Van Gaal LF Effect of sibutramine on weight maintenance after weight loss: a randomised trial. STORM Study Group. Sibutramine Trial of Obesity Reduction and Maintenance Lancet 2000 356 2119 2125 10.1016/S0140-6736(00)03491-7 11191537

[B24] Yuan G Al-Shali KZ Hegele RA Hypertriglyceridemia: its etiology, effects and treatment CMAJ 2007 176 1113 1120 17420495 10.1503/cmaj.060963 PMC1839776

