# Supplementary material for: The weight lowering effect of sibutramine and its impact on serum lipids in cardiovascular high risk patients with and without type 2 diabetes mellitus - an analysis from the SCOUT lead-in period
Source: BMC Endocr Disord. 2010 Feb 26;10:3. doi: 10.1186/1472-6823-10-3 (PMC2848038; doi:10.1186/1472-6823-10-3)
Supplement: Additional file 1 — Estimated effect of variables from Table 1on lipid changes according to multivariable regression analyses. The additional file contains the results from the multivariable regression analysis examining the effects on lipid changes when controlling for all variables listed in Table 1 [file 1472-6823-10-3-S1.DOC]

Additional File 1: Estimated effect of variables from Table 1 on lipid changes according to multivariable regression analyses

| **Screening information on:** | **LDL-C** | **P** | **HDL-C** | **P** | **VLDL-C** | **P** | **Total  cholesterol** | **P** | **Trig** | **P** |
| --- | --- | --- | --- | --- | --- | --- | --- | --- | --- | --- |
| **Sex (male)** | **-0.069** | **(0.0012)** | **-0.0206** | **(<.0001)** | **-0.048** | **(<.0001)** | **-0.128** | **(<.0001)** | **-0.092** | **(<.0001)** |
| **Age (years)** | **-0.002** | **(0.0383)** | 0.0002 | (0.3750) | -0.0003 | (0.5305) | **-0.003** | **(0.0294)** | -0.002 | (0.1081) |
| **Weight (kg)** | -0.0003 | (0.7270) | -0.0002 | (0.5061) | **0.001** | **(0.0120)** | 0.0007 | (0.5010) | **0.003** | **(0.0056)** |
| **BMI (kg/m^2^)** | **0.006** | **(0.0478)** | -0.0003 | (0.6735) | 0.001 | (0.4864) | **0.008** | **(0.0221)** | 0.003 | (0.2794) |
| **LDL-C (mmol/L)** | **-1.331** | **(<.0001)** | **-0.136** | **(0.0325)** | -0.045 | (0.6760) | -0.567 | (0.0625) | -0.282 | (0.3130) |
| **HDL-C (mmol/L)** | **-1.000** | **(0.0001)** | **-0.328** | **(<.0001)** | -0.118 | (0.2796) | -0.506 | (0.0985) | -0.438 | (0.1193) |
| **VLDL-C (mmol/L)** | -0.375 | (0.0830) | -0.069 | (0.1881) | **-0.483** | **(<.0001)** | 0.153 | (0.5449) | **1.350** | **(<.0001)** |
| **TC (mmol/L)** | **0.988** | **(0.0002)** | **0.133** | **(0.0358)** | 0.052 | (0.6328) | 0.230 | (0.4500) | 0.298 | (0.2854) |
| **Triglycerides (mmol/L)** | **-0.277** | **(0.0312)** | -0.032 | (0.3031) | 0.007 | (0.9021) | **-0.341** | **(0.0229)** | **-1.096** | **(<.0001)** |
| **Waist (10 cm)** | -0.002 | (0.8407) | **-0.007** | **(0.0086)** | **0.014** | **(0.0009)** | -0.001 | (0.9376) | **0.025** | **(0.0265)** |
| **Hip (10 cm)** | -0.011 | (0.2908) | 0.001 | (0.8224) | **-0.008** | **(0.0445)** | -0.022 | (0.0601) | **-0.032** | **(0.0037)** |
| **Diastolic BP (10 mmHg)** | **0.033** | **(0.0004)** | 0.004 | (0.0994) | 0.001 | (0.8112) | **0.035** | **(0.0011)** | -0.002 | (0.8166) |
| **Systolic BP (10 mmHg)** | -0.003 | (0.6043) | -0.001 | (0.6103) | 0.004 | (0.1209) | 0.0018 | (0.7986) | **0.014** | **(0.0320)** |
| **Pulse (b.p.m.)** | **0.017** | **(0.0134)** | -0.002 | (0.1261) | **0.008** | **(0.0073)** | **0.024** | **(0.0021)** | **0.024** | **(0.0009)** |
| **History of:** |  |  |  |  |  |  |  |  |  |  |
| **Diabetes*** | **-0.060** | **(0.0012)** | 0.001 | (0.7592) | **0.030** | **(<.0001)** | -0.029 | (0.1706) | **0.065** | **(0.0010)** |
| **Hyperlipidemia** | **0.095** | **(<.0001)** | 0.001 | (0.8873) | 0.014 | (0.0921) | **0.102** | **(<.0001)** | 0.008 | (0.7039) |
| **Smoking** | 0.029 | (0.1956) | -0.009 | (0.0908) | **0.042** | **(<.0001)** | **0.051** | **(0.0472)** | **0.087** | **(0.0002)** |
| **Drinking** | **-0.053** | **(<.0001)** | 0.002 | (0.5574) | -0.006 | (0.2553) | **-0.060** | **(0.0001)** | -0.019 | (0.1927) |
| **Use of:** |  |  |  |  |  |  |  |  |  |  |
| **Fibrates** | -0.004 | (0.8658) | -0.0005 | (0.9349) | **0.02** | **(0.0222)** | 0.03 | (0.2726) | **0.081** | **(0.0016)** |
| **Betablockers** | 0.002 | (0.9029) | **-0.020** | **(<.0001)** | **0.037** | **(<.0001)** | 0.026 | (0.1294) | **0.103** | **(<.0001)** |
| **Statins** | **-0.300** | **(<.0001)** | -0.004 | (0.3581) | -0.013 | (0.0998) | **-0.312** | **(<.0001)** | -0.015 | (0.4599) |
| **Change in:** |  |  |  |  |  |  |  |  |  |  |
| **BMI (-1kg/m^2^)*** | **-0.106** | **(<.0001)** | **-0.013** | **(<.0001)** | **-0.072** | **(<.0001)** | **-0.201** | **(<.0001)** | **-0.180** | **(<.0001)** |

‘10’ denotes change per 10 unit increase. P values are shown within ( ). We also controlled for History of: coronary artery disease (CAD), congestive heart failure (CHF), peripheral artery disease (PAD), Stroke, Hypertension, AMI and use of ACEi: angiotensin converting enzyme inhibitor.*Interaction was found between reduction in BMI (-1kg/m^2^) and diabetes. Significant values on a 95% significance level are highlighted with bold. 13 patients with unknown risk category were excluded
